# Controlling Johne's disease: vaccination is the way forward

**DOI:** 10.3389/fcimb.2015.00002

**Published:** 2015-01-21

**Authors:** John P. Bannantine, Adel M. Talaat

**Affiliations:** ^1^Infectious Bacterial Diseases, National Animal Disease Center, United States Department of Agriculture - Agricultural Research ServiceAmes, IA, USA; ^2^Department of Pathobiological Sciences, University of WisconsinMadison, WI, USA

**Keywords:** Johne's disease, vaccines, Mycobacterium, paratuberculosis, veterinary medicine

*M. avium* subspecies *paratuberculosis*, hereafter referred to as *MAP*, is a significant veterinary pathogen that causes Johne's disease in ruminants, including cattle, sheep, and goats. This chronic intestinal disease is distributed worldwide and exacts a heavy economic toll on animal producers. For example, the dairy industry incurs substantial economic losses due to reduced milk production, premature culling, and reduced slaughter value (Raizman et al., 2009). It takes years for clinical signs to appear in animals after initial infection. The bacterium is shed in high numbers in the feces during this clinical phase of disease. Transmission is by ingestion of the bacterium while grazing on pastures contaminated by this shedding process. Milk, passed from the infected dam to the daughter, has also been shown as a transmission route (Stabel, 2008). To best combat this chronic infection, vaccination has the promise to reduce economic losses and control Johne's disease. In the conception of this eBook for Frontiers in Cellular and Infection Microbiology, we solicited communications describing technologies and approaches to immunize animals against Johne's disease.

Among the first large-scale vaccine trials for Johne's disease began in the early 1990s using killed whole cells in oil injected into cattle (Kormendy, 1994; Wentink et al., 1994). Those trials showed vaccination was useful to reduce shedding of the bacteria in the feces, thus potentially reducing cow-to-calf transmission, but was ineffective at preventing infection. Since that time, experiments have evaluated extracts of the bacteria and live cells in all hosts including sheep, goats, deer, and cattle. Furthermore, the strain of *MAP* used has varied greatly in those studies. Then in 2007, an effort was made to standardize the challenge models used to test new vaccines for combating Johne's disease (Hines et al., 2007). The parameters outlined for the goat model in that article were used to test the best available live attenuated candidates (Hines et al., 2014) as described in this eBook compilation.

The genome sequence and re-sequence of *MAP* (Li et al., 2005; Wynne et al., 2010) has greatly expanded our knowledge about proteins predicted to be located in the mycobacterial envelope through annotation and bioinformatic analyses. Even now, most of what is known can be obtained only from annotation predictions and computer modeling algorithms that suggest protein locations within a cell (Yu et al., 2010). A perfect example of the power of genomics was demonstrated a few years later when libraries of transposon mutants and transcriptomic studies were reported by several groups working with *MAP* (Shin et al., 2006; Wu et al., 2006, 2007; Janagama et al., 2010). Furthermore, genomics provided an important resource for targeted construction of live-attenuated vaccine (LAV) candidates, which is the focus of several articles in this eBook. It is noteworthy that despite a focus on LAV strategies, we do not discount efforts directed toward the development of other vaccine technologies such as subunit or vectored vaccines (Hoek et al., 2010; Faisal et al., 2013; Thakur et al., 2013). Although these technologies are still under development, they could provide effective vaccines in the future.

Live attenuated vaccine strains of *MAP* are thought to be the best approach for vaccination against Johne's disease. Although traditional inactivated and LAV candidates cannot satisfy the DIVA approach, they will completely stimulate both cell-mediated and humoral immune responses (Park et al., 2011; Faisal et al., 2013). Numerous studies have suggested the role of cell-mediated immunity to provide protective responses against *MAP* infection, as suggested before in both the murine (Ghosh et al., 2014; Settles et al., 2014) and bovine models (Stabel et al., 2011) of Johne's disease. A partial analysis of the generated immune responses following immunization with novel LAV candidates is reported in this eBook (Hines et al., 2014) as part of a large scale vaccine trial. Additional analyses on the T cell populations and specific antigens detected will be forthcoming (Bannantine et al., unpublished).

To summarize the contributions to this research topic, the opening article reviews efforts by the Johne's disease research community to test available LAV strains of *MAP* through a series of three gated trials (Bannantine et al., 2014b). This trial series has been a coordinated international, multi-institutional effort that spanned several years. Many new ideas and retrospective approaches have emerged from this unprecedented effort. These aspects have been captured in this research topic. Details surrounding the construction of the attenuated mutants are also included as well as a list of the lessons learned from this integrated study. The next two articles describe a knockout mutant of *relA* (Park et al., 2014) and a library of transposon mutants (Rathnaiah et al., 2014). Both approaches are useful for generating live attenuated strains. The next three articles (Bannantine et al., 2014a; Hines et al., 2014; Lamont et al., 2014) describe in detail the three phases of the vaccine project summarized in the opening article. The remaining articles highlight immunological evaluation of subunit vaccines (Gurung et al., 2014), production of subunit vaccines using heterologous hosts (Johnston et al., 2014) and finally, take a unique look at the importance of post-translational modifications for vaccine design, with a highlight on glycoproteins (Facciuolo and Mutharia, 2014).

## Conflict of interest statement

The authors declare that the research was conducted in the absence of any commercial or financial relationships that could be construed as a potential conflict of interest.
